# Phylogenetic Analysis of Glucosyltransferases and Implications for the Coevolution of Mutans Streptococci with Their Mammalian Hosts

**DOI:** 10.1371/journal.pone.0056305

**Published:** 2013-02-14

**Authors:** Silvia Argimón, Alexander V. Alekseyenko, Rob DeSalle, Page W. Caufield

**Affiliations:** 1 Department of Cariology and Comprehensive Care, New York University College of Dentistry, New York, New York, United States of America; 2 Center for Health Informatics and Bioinformatics, New York University School of Medicine, New York, New York, United States of America; 3 Sackler Institute for Comparative Genomics, American Museum of Natural History, New York, New York, United States of America; Centers for Disease Control & Prevention, United States of America

## Abstract

Glucosyltransferases (Gtfs) catalyze the synthesis of glucans from sucrose and are produced by several species of lactic-acid bacteria. The oral bacterium *Streptococcus mutans* produces large amounts of glucans through the action of three Gtfs. GtfD produces water-soluble glucan (WSG), GtfB synthesizes water-insoluble glucans (WIG) and GtfC produces mainly WIG but also WSG. These enzymes, especially those synthesizing WIG, are of particular interest because of their role in the formation of dental plaque, an environment where *S. mutans* can thrive and produce lactic acid, promoting the formation of dental caries. We sequenced the *gtfB*, *gtfC* and *gtfD* genes from several mutans streptococcal strains isolated from the oral cavity of humans and searched for their homologues in strains isolated from chimpanzees and macaque monkeys. The sequence data were analyzed in conjunction with the available Gtf sequences from other bacteria in the genera *Streptococcus*, *Lactobacillus* and *Leuconostoc* to gain insights into the evolutionary history of this family of enzymes, with a particular emphasis on *S. mutans* Gtfs. Our analyses indicate that streptococcal Gtfs arose from a common ancestral progenitor gene, and that they expanded to form two clades according to the type of glucan they synthesize. We also show that the clade of streptococcal Gtfs synthesizing WIG appeared shortly after the divergence of viviparous, dentate mammals, which potentially contributed to the formation of dental plaque and the establishment of several streptococci in the oral cavity. The two *S. mutans* Gtfs capable of WIG synthesis, GtfB and GtfC, are likely the product of a gene duplication event. We dated this event to coincide with the divergence of the genomes of ancestral early primates. Thus, the acquisition and diversification of *S. mutans* Gtfs predates modern humans and is unrelated to the increase in dietary sucrose consumption.

## Introduction

*Streptococcus mutans* is a member of the indigenous human oral biota, of worldwide distribution [1]. It largely contributes to the formation of the biofilm known as dental plaque through the production of extracellular glucans from dietary sucrose. Glucans mediate the attachment of bacteria to the tooth surface [2], [3] and to other members of the oral biota [4], [5], thus promoting biofilm development. In this environment *S. mutans* produces mainly lactic acid as the byproduct of the metabolism of sucrose and other carbohydrates. This, in turn, can lead to the erosion of tooth enamel and dentin, and the emergence of dental caries [1].

Glucans are high molecular weight d-glucose polymers synthesized by extracellular enzymes called glucosyltransferases (more generally known as glucansucrases, EC 2.4.1.5). Glucosyltransferases (Gtfs) belong to the glycosidase family 70, and catalyze the transfer of glucosyl units from the cleavage of sucrose to a growing α-glucan chain, employing only the energy released from splitting of the glycosidic bond [6]. Gtfs are prevalent among the homofermentative lactic acid bacteria, including the oral species of *Streptococcus* and *Lactobacillus*, as well as in species of *Leuconostoc* and *Lactobacillus* isolated from fermented foods or the environment [7]. The number of glucosyltransferase genes found in a given species may vary from one to four [8], with each enzyme synthesizing a different type of glucan depending on the nature of the glucosidic bond. Water-soluble glucans (WSG), also known as dextran, are rich in α-1,6 glucosidic linkages, while a higher content of α-1,3 glucosidic linkages is characteristic of water-insoluble glucans (WIG), also known as mutan [9].

In *S. mutans* the synthesis of glucans is catalyzed by three Gtfs: GtfB, GtfC and GtfD (also known as Gtf-I, Gtf-SI and Gtf-S, respectively; [10]). GtfD synthesizes WSG in a primer-dependent manner [11], i.e., it requires a preformed dextran for polymerization to occur at a near maximum rate. GtfB and GtfC are encoded by highly homologous genes that are tandemly arranged, likely as the result of a gene duplication event [12]. Both Gtfs synthesize WIG in a primer-independent manner, although GtfC can also synthesize WSG [13].

*S. mutans* can be found in virtually all dentate humans, not just individuals with dental caries [1]. It is acquired in infancy mostly from the mother, and its colonization of the oral cavity is stable [14]. Glucans, particularly WIG, play a key role in *S. mutans* sucrose-dependent adhesion [15] and biofilm formation [16]. This lends itself to the interpretation that, when sucrose became a significant part of the human diet, the presence of *gtf* genes could have conferred a selective advantage to the oral bacteria harboring them [1]. In this scenario, the acquisition of *gtf* genes through horizontal gene transfer between oral species, or by gene duplication followed by functional divergence, would be advantageous. Selection pressure would maintain multiple *gtf* genes if they independently contributed to fitness. The correlation between sucrose metabolism within the dental biofilm and the incidence of dental caries, have positioned the glucosyltransferases in the spotlight of *S. mutans* virulence. It was therefore of interest to study the acquisition of the glucosyltransferase genes by *S. mutans* in the context of the evolution of this family of enzymes.

## Materials and Methods

### Bacterial Strains and DNA Isolation

A total of 18 oral strains were included in this study. Thirteen plasmid-containing strains of *S. mutans* in our collection were previously collected from saliva or plaque from caries-active subjects from five different continents [17]. Eight oral isolates of mutans streptococci were obtained from African chimpanzees (*Pan troglodytes*) at the request of PC by Mark Achtman from Dr. Lawrence Mugisha, Department of Wildlife and Animal Resources Management, Kampala, Uganda. The samples were taken as part of a previous study by a group from the Max Planck Institute in Berlin. The biopsy samples and dental scrapings were collected in full accordance with set guidelines by the International Primatological Society, Pan African Sanctuaries Alliance (PASA) and Standard Operating Procedures by the Chimpanzee Sanctuary & Wildlife Conservation Trust (CSWCT) that practices the highest welfare standards of the chimpanzees in the sanctuary. The biopsy samples were collected by Dr. Lawrence Mugisha during the annual health checks as part of preventive medical care under general anesthesia. In addition, the sample collection was approved by Uganda Wildlife Authority (UWA) and UNCST ref no: NS71 that reviews the ethical procedures with guidance from National Institutional Board Review (IBR). Import and export permits from CITES (permit nos: E-0437/08 and 001944 respectively were obtained to ship the biological samples (biopsies) for analysis. Analysis of the 16S rRNA gene sequences showed that they were closely related to *S. mutans* (not shown), and four distinct genotypes were identified by chromosomal DNA fingerprinting as previously described [17] and used for this study. A novel species, *Streptococcus troglodytae*, was recently proposed for oral strains of mutans streptococci isolated from chimpanzees and closely related to *S. mutans* [18]. We adopted this name for our strains based on the similarity of the 16S rRNA gene sequences (not shown). *Streptococcus macacae* strain NCTC 11558 was isolated from the oral cavity of a macaque monkey (*Macaca fascicularis*) by Beighton et al. (1984) [19], and was purchased from the American Type Culture Collection (ATCC 35911). Strains were streaked onto solid Todd-Hewitt medium and grown anaerobically for three days. Cell lysis and chromosomal-DNA isolation were performed as previously described [20], [21].

### Cloning and Sequencing of Glucosyltransferase Genes

For the human strains, PCR primers specific for each of the *S. mutans gtfB*, *gtfC* and *gtfD* genes were designed based on sequences available at the NCBI database (Table S1). For the chimpanzee and macaque strains, primers were designed based on the draft genome assemblies generated in-house as part of an ongoing study (data not published). The *gtfB* and *gtfC* genes were PCR amplified separately with Elongase (Invitrogen, Carlsbad CA), cloned into the pCR-XL-TOPO vector (Invitrogen), and transformed into chemically competent *E. coli* TOP10 cells, all following manufacturer instructions. Colonies were tested for the presence of the *gtf* amplicon by white/blue screening and enzymatic restriction digestion with *Mlu*I. The positive clones were sequenced in both directions at Genewiz (South Plainfield, NJ), with primers M13-F(-20), M13-R and the primers listed in Table S1. Sequence reads were assembled with using the Sequence Assembler module in Bionumerics version 6.0 (Applied Maths, Belgium). The nucleotide and predicted amino acid sequences were deposited into GenBank (accession numbers JX072971-JX073024). During the course of this study, an assembly of the *S. macacae* strain NCTC 11558 became available (GenBank AEUW00000000). The *gtfC* and *gtfD* sequences were identical to the ones obtained by us, but the *gtfB* sequence available at NCBI contained a 195-nt deletion coinciding with the glucan binding YG repeats.

Over 140 available nucleotide and amino acid sequences of glucosyltransferases/glucansucrases from *Streptococcus*, *Lactobacillus*, *Leuconostoc*, *Oenococcus* and *Weisella* species were obtained from the NCBI database. To obtain a more manageable number of sequences we clustered similar sequences with BlastClust (cutoff 97%). Partial, inactive or genetically engineered sequences were then manually removed, to obtain a collection of 69 full-length glucosyltransferase/glucansucrase sequences representing 32 species (Table S2), in addition to the sequences from this study.

### Phylogenetic Inference

**Figure 1:**
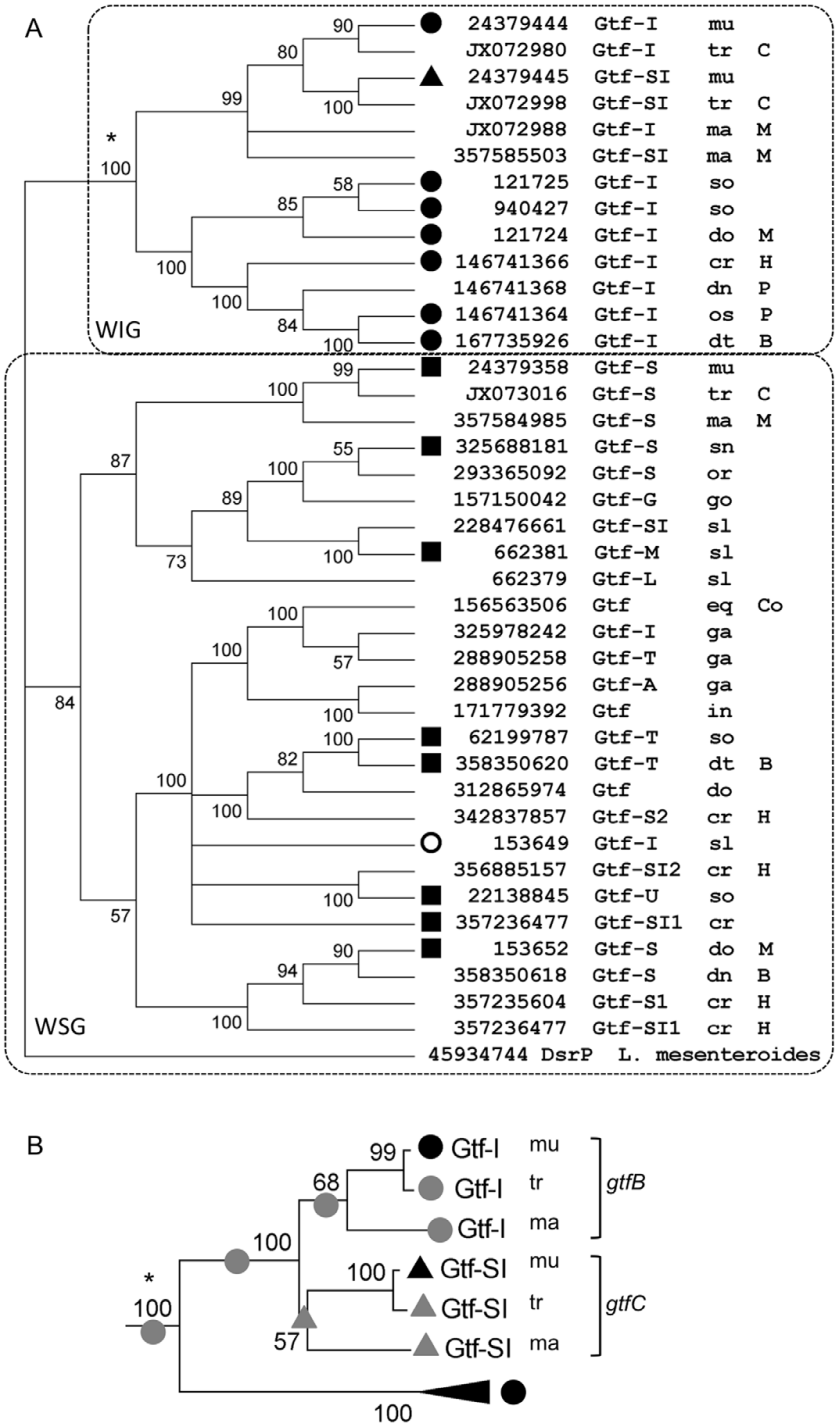
Phylogenetic analysis of streptococcal glucosyltransferases. A) Maximum likelihood consensus tree of 39 streptococcal glucosyltransferases based on the amino acid sequence of the catalytic domain. Node values indicate bootstrap support from 500 replicates. Branches with less than 50% support were collapsed. Inset: tree topology for the *S. mutans* water-insoluble glucan cluster based on the full length amino acid sequence. Biochemical data on the type of glucan is indicated as circles for WIG, squares for WSG, and triangles for gtfs that synthesize both WIG and WGS. Bacterial species are indicated as follows: cr = *S. criceti*; dt = *S. dentirousetti*; dn = *S. dentisuis*; do = *S. downei*; eq = *S. equinus*; ga = *S. gallolyticus*; go = *S. gordonii*; in = *S. infantarius*; ma = *S. macacae*; mu = *S. mutans*; or = *S. oralis*; os = *S. orisuis*; sl = *S. salivarius*; sn = *S. sanguinis*; so = *S. sobrinus*; tr = *S. troglodytae*. The strains were isolated from human subjects unless indicated as follows: B = bat; C = chimpanzee; Co = cow; H = hamster; M = macaque; P = pig. B) Detail of the tree topology for the WIG cluster of *S. mutans* Gtfs based on the full length amino acid sequence. Reconstruction of character states is indicated by gray circles and triangles.

Phylogenetic analyses were conducted on both the nucleotide coding sequences and the predicted amino acid sequences aligned with MAFFT v6.864b [22] using the L-INS-i strategy for accuracy. Maximum likelihood (ML) was used as the optimality criterion, and optimal nucleotide and amino acid substitution models were determined with MEGA5 [23] and PROTTEST v2.4 [24] and the likelihood-ratio method [25]. Tree searches were conducted with both PAUP 4.0a123 [26] and MEGA 5 under the Tamura-Nei 93 nucleotide substitution model (TN93; [27]), or either the Whelan and Goldman (WAG; [28]) or the Jones, Taylor and Thornton (JTT; [29]) amino acid substitution models. Substitution models were combined with empirical estimates of nucleotide/amino acid frequencies and, a gamma distributed among-sites rate variation, and an estimate of the proportion of invariant sites. Positions containing gaps were excluded from the analysis. Bootstrapping was performed with 500 replicates. The dataset for Figure S1, was composed of 66 glucosyltransferase/glucansucrase sequences from bacteria in the genera *Streptococcus*, *Lactobacillus, Leuconostoc, Oenococcus and Weisella.* The dataset for Figure 1 was composed of 39 streptococcal glucosyltransferase sequences representing 16 species. The tree was constructed based on the predicted amino acid sequence of the conserved catalytic domain of Gtfs (positions 166–934 in *S. mutans* GtfB) and rooted with the dextransucrase DsrP from *L. mesenteroides*, chosen as an outgroup on the basis of its position in the tree presented in Figure S1. A consensus tree was generated by collapsing the branches with less than 50% bootstrap support, and thus the branch lengths are not shown.

Bayesian estimate of the phylogeny and node ages were obtained by Markov chain Monte Carlo sampling with a strict clock as implemented in BEAST 1.7.1 [30]. Our dataset for the Bayesian phylogeny included the GtfB, GtfC and GtfD sequences from 13 *S. mutans* human strains, 4 *S. mutans* strains from chipanzees and 1 *S. macacae* strain obtained in this study, the WIG Gtf sequences from *S. downei* (GI 121724), *S. criceti* (GI 146741366) and *S. orisuis* (GI 146741364) strains isolated from macaque, hamster and pig, respectively (Table S2), as well as the WSG Gtf sequences from *S. dentirousetti* (GI 358350620), *S. downei* (GI 153653) and *S. criceti* (GI 357235604) isolated from bat, macaque and hamster, respectively. Two sequences served as an outgroup for this dataset: the dextransucrase DsrP from *Leuconostoc mesenteroides* (GI 45934744), and the predicted glucansucrase from *Weisella cibaria* (GI 332638569). Phylogeny inference was conducted both on nucleotide and on amino acid sequences with the same calibration points, and the results were compared. Nucleotide sequences were aligned based on the predicted amino acid sequences with TranslatorX choosing the MAFFT method. The amino acid sequences were aligned using MAFFT v6.864b. A normal prior distribution was applied on the ages of the calibration points, with a mean of 7.5±1.1 My for divergence time of the human lineage from the chimpanzee lineage, a mean of 31±4 My for the divergence from the Old World monkeys (macaque) [31], and a mean of 104.5±5.5 My for the divergence time of placental mammals [32]. Two independent runs of 10 million iterations (subsampling every 1000^th^ iteration) were performed for each analysis, using a Tn93+Γ+I and a WAG+Γ+I substitution model for the nucleotide and amino acid data, respectively, a Yule tree prior [33], a gamma distribution for the clock rate prior, and the default options for all other prior and operator settings. The convergence of the MCMC chains was assessed by inspection of the trace plots and the effective sample sizes using Tracer 1.5 [34]. Maximum clade credibility trees were annotated using TreeAnnotator [34] with a posterior probability limit of 0.5 and mean node heights, and visualized in FigTree v1.3.1 (http://tree.bio.ed.ac.uk/software/figtree/).

### Detection of Selection and Protein Modeling

The signatures of selection operating on the *gtfB, gtfC* and *gtfD* genes were detected using the single-likelihood ancestor counting (SLAC), fixed effects likelihood (FEL) and random effects likelihood (REL) methods [35] implemented in the HyPhy package [36] available at the Datamonkey webserver [37], [38]. This dataset was composed of the 18 nucleotide sequences for each *gtf* gene obtained in this study from human, chimpanzee and macaque strains. Individual *gtfB*, *gtfC* and *gtfD* sequence alignments were generated based on the predicted amino acid sequences with TranslatorX [39]; http://translatorx.co.uk/) and inspected manually. The alignments were screened for the presence of recombination with the GARD method [40] previous to the SLAC, FEL, and REL analyses.

Models of the 3D structure of proteins GtfB (residues 219 to 1062) and GtfD (residues 230 to 1091) were built with Swiss-Model [41], [42] based on the crystal structure of the catalytic domain of *S. mutans* Gtf-SI from strain MT8148 (Protein Databank accession code 3AIE; [43]). The resulting structures were viewed with Polyview-3D [44].

### Functional Divergence

Types I and II functional divergence between *S. mutans* GtfB and GtfC were estimated with DIVERGE2 [45], [46] based on an alignment of Gtf sequences from human, chimpanzee and macaque isolates and a Neighbor Joining tree constructed with MEGA5. Type I and type II functional divergence coefficients (θ_I_ and θ_II_, respectively) between the GtfB and GtfC clusters were calculated with a maximum-likelihood method and their significance was assessed with a likelihood ratio test (LRT). The amino acid residues that were critical for functional divergence were identified as those with a posterior probability (P) of functional divergence above the cutoff value of P>0.85.

## Results

### A Tree Based Classification of Gtf Proteins and Genes

Genes encoding glucosyltransferases, more generally known as glucansucrases, have been found thus far in five different genera of lactic-acid bacteria: *Streptococcus*, *Lactobacillus*, *Leuconostoc*, *Weisella* and *Oenococcus*. The phylogeny of the 66 glucansucrases in our dataset (Figure S1 and Table S2) suggests a monophyletic relationship among the streptococcal enzymes, regardless of the type of glucan synthesized. Glucansucrases from *Lactobacillus* and *Leuconostoc* species, on the other hand, form polyphyletic groups.

### Correlation of Divergence of Gtfs with their Function

Streptococcal glucosyltransferases synthesize WSG (dextran) with a predominance of α-1,6 glucosidic linkages, WIG (mutan) with a high proportion of α-1,3 glucosidic linkages and, in the case of *S. mutans* GtfC, both types of glucans are synthesized [9]. A more detailed reconstruction of the phylogeny of 39 glucosyltransferases representing 16 *Streptococcus* species is presented in Figure 1A. Streptococcal glucosyltransferases clustered by the type of glucan they synthesize. The first clade contained the Gtfs that synthesize WIG, including *S. mutans* GtfB (Gtf-I) and GtfC (Gtf-SI). The phylogenetic relationships between *S. macacae, S. troglodytae* and *S. mutans* GtfB and GtfC were not resolved with this approach. A phylogeny inferred from the full-length Gtf amino acid sequences showed that the GtfB and GtfC sequences form two sister groups, with bootstrap support values only slightly higher than 50% (Figure 1B). The most parsimonious reconstruction of character states presented in Figure 1B leads us to believe that the most likely common ancestor to GtfB and GtfC is a WIG-synthesizing Gtf, thereby suggesting that the ability of GtfC to synthesize WSG was likely acquired after the gene duplication event. It is also noteworthy that all the glucosyltransferases from the WIG clade belong to *Streptococcus* species from the mutans group [47].

The second clade contains the Gtfs that synthesize WSG. This group also includes Gtf-L and Gtf-I from *S. salivarius*, which synthesize WIG. These two enzymes present peculiarities that may explain this fact. On one hand Gtf-L synthesizes a glucan with equal proportions of α-1,3 and α-1,6 glucosydic linkages [8]. On the other hand, Gtf-I (encoded by *gtfJ*) is acceptor-dependent, that is the enzyme requires a primer glucan for activity [8], and is likely to have arisen from a gene duplication event since *gtfJ* is immediately upstream from *gtfK*, encoding acceptor-dependent Gtf-S that synthesizes WSG.

There is also some correlation within the WSG group with the subdivision of the *Streptococcus* genus into groups based on the 16S rRNA gene sequence [47]. For example, a very robust clade is composed of all the Gtf sequences from species belonging to the bovis group, which are common inhabitants of the intestinal flora. Concordantly, these strains have been isolated from sites other than the oral cavity (Table S2).

### Role of Natural Selection in the Evolution of *S. mutans* Gtfs

The predominant role of WIG over WSG in sucrose-dependent adhesion [15] and *S. mutans* cariogenicity [48] could lead to the assumption that the acquisition and diversification of WIG-synthesizing Gtfs in *S. mutans* could reflect the increase in sucrose consumption by humans. We found genes homologous to *S. mutans gtfB*, *gtfC* and *gtfD* in the draft genomes of *S. troglodytae and S. macacae* strains isolated from chimpanzees and macaque, respectively, strongly suggesting that the acquisition of WIG-synthesizing Gtfs took place well before the origin of humans.

Given the significance of Gtfs in the formation of the dental biofilm and in cariogenesis it was of interest to investigate whether selective pressure affecting *S. mutans* sucrose metabolism was driving the functional divergence of Gtfs. We analyzed the presence of signatures of selection on Gtf proteins based on the relative rates of synonymous and non-synonymous substitutions with the integrative analysis that combines the SLAC, FEL and REL methods as implemented in Datamonkey. Evidence of positive selected sites was found with FEL for all three Gtfs, but they were not supported by the REL and SLAC analyses. On the other hand, 60, 43 and 29 negatively selected sites (i.e., under purifying selection) were identified by all three methods for GtfB, GtfC and GtfD, respectively. Fifteen negatively selected amino acids were conserved between GtfB and GtfC, while only one amino acid under negative selection was common to the GtfB and GtfD or GtfC and GtfD pairs.

**Figure 2:**
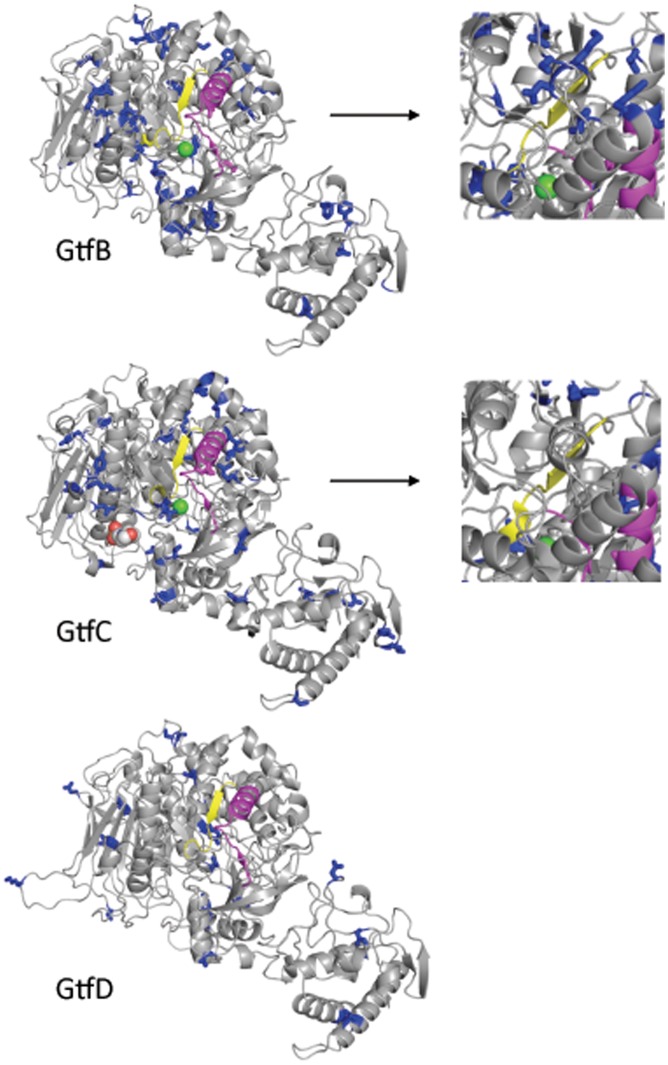
Models of the 3D structure of the catalytic domain of *S. mutans* Gtfs. Blue: amino acids under negative selection (side chains shown). Yellow: active site. Magenta: Gtf-P1 region [69]. Green sphere: Ca^2+^ ion. Right panels: Detail of a different view of the region surrounding the active site of GtfB and GtfC.

As a result of the conservation of function in catalytic domains of proteins, most of the amino acids predicted to be under purifying selection were located in the catalytic domains of the three proteins. However, there are some interesting departures from this general expectation based on location of the catalytic domain. Figure 2 shows that the catalytic domains of GtfB and GtfC are under stronger negative selection than the catalytic domain of GtfD. In addition the active site of GtfB appears to be tightly surrounded by negatively selected amino acids, even more so than that of GtfC.

### Gene Duplications and Timing of Functional Divergence of *S. mutans* Gtfs

**Figure 3:**
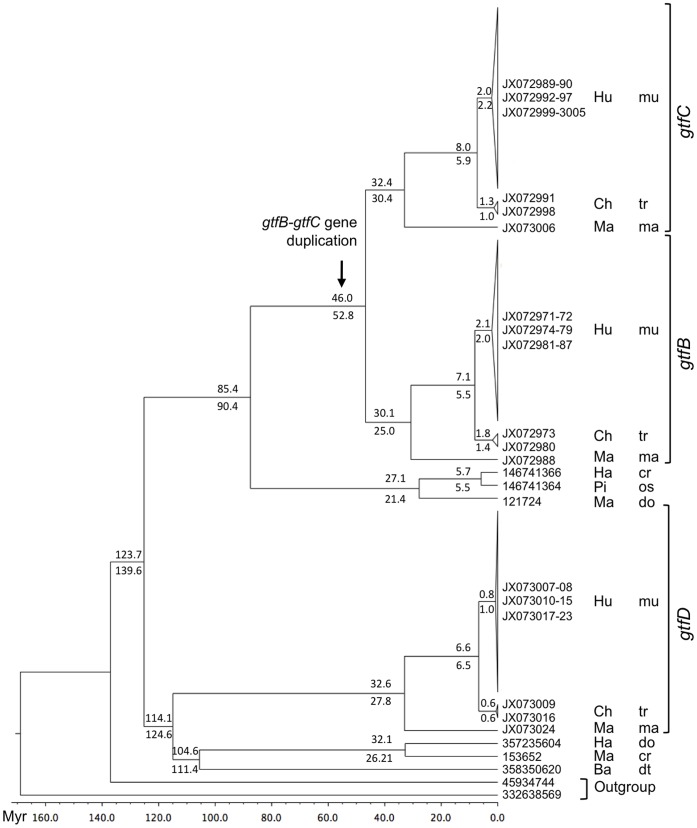
Bayesian phylogeny of streptococcal Gtfs. Values represent mean node ages obtained from either the nucleotide (top) or the amino acid (bottom) sequences, with the node calibration from [31]. The substitution models were TN93+Γ+I and WAG+Γ+I for nucleotide and amino acid data, respectively. The scale at the bottom represents time before present in millions of years. Bacterial species are indicated as follows: cr = *S. criceti*; dt = *S. dentirousetti*; do = *S. downei*; ma = *S. macacae*; mu = *S. mutans*; os = *S. orisuis*; tr = *S. troglodytae*. Strains isolated from Hu = human; Ch = chimpanzee; Ma = macaque; Ha = hamster; Pi = pig; Ba = bat.

Gene duplication is a key mechanism in the evolution of gene diversity. After duplication, one or both duplicates can accumulate amino acid changes, thereby promoting functional divergence through the action of natural selection. To estimate the time of the *gtfB*-*gtfC* gene duplication event in *S. mutans* we analyzed a dataset including *gtfB*, *gtfC* and *gtfD* sequences from *S. mutans*, *S. troglodytae*, and *S. macacae* strains with a Bayesian method. The posterior probability distribution for the *gtfB*-*gtfC* divergence time is characterized by a mean of 46.0 Mya (95% CI = 42.0–50.0 Mya) for the nucleotide data or 52.8 Mya (95% CI = 47.1–58.0 Mya) for the amino acid data. Our results support that the *gtf*B-*gtf*C gene duplication event occurred around the time of divergence of primates (Figure 3). In addition, the GtfB and GtfC sequences from humans, chimpanzees and macaque formed two well-resolved sister groups with posterior probabilities of 1, a phylogenetic relationship that was not resolved with the maximum likelihood tree of all streptococcal Gtf presented in Figure 1.

Even though both GtfB and GtfC synthesize WIG, there are several differences between the two enzymes, most notably that GtfC can also synthesize WSG [13]. GtfB and GtfC also exhibit different sucrose-dependent binding properties [49], [4], [50] and different activities in a mixed oral biofilm model [51]. This suggests that, despite their high conservation (74.5% sequence identity), they have diverged enough to gain different functions. Functional divergence of duplicated genes can result in different evolutionary rates at certain amino acids, known as type I functional divergence [52], [45], or in site-specific property shifts, typically represented by a radical shift in amino acid property and known as type II functional divergence [45], [53]. We estimated the functional divergence between GtfB and GtfC using DIVERGE2 [46]. The coefficient of type I functional divergence between GtfB and GtfC was significantly different from zero (θ_I_ML = 0.301±0.039; LRTθ_I_ = 58.258, p<<0.005), indicating that the site-specific evolutionary rate differs between the GtfB and GtfC clades. On the other hand, DIVERGE2 failed to identify site-specific amino acid substitutions that represent a change in the physicochemical properties and that are fixed in each clade (coefficient of type II divergence θ_II_ = 0.075±0.019).

**Figure 4:**
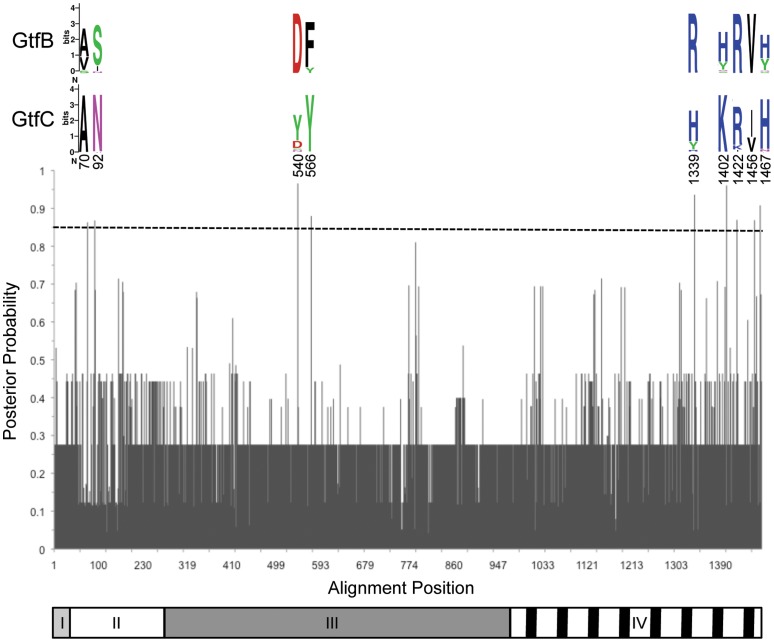
Site specific profile of type I functional divergence posterior probability. Logos are shown for positions predicted to be critical for type I functional divergence between GtfB and GtfC (cutoff P>0.85). Residues are color coded by biochemical property and heights represent their relative frequency at each site. The Gtf protein domains are represented below the graph. I) signal peptide, II) N-terminal variable region, III) catalytic domain, IV) glucan binding domain.

The amino acid residues responsible for type I functional divergence were identified based on the site-specific posterior probability (Figure 4). From the nine sites with a posterior probability of type I divergence between GtfB and GtfC larger than 0.85, four (positions 540, 1339, 1442 and 1456 in our alignment) were completely conserved in GtfB but variable in GtfC, and four (positions 70, 92, 566 and 1042) were completely conserved in GtfC but variable in GtfB. The remaining residue (position 1467) was mostly conserved in GtfC but variable in GtfB. In addition, two residues were located in the N-terminal variable region of these proteins, two were located in the catalytic domain and four were located in the glucan binding domain (GBD). The two type I residues located in the catalytic domain were outside (but close) of the active site and the 19-amino acid Gtf-P1 region, both of which are completely conserved between the GtfB and GtfC clades (not shown). Only the crystal structure of the catalytic domain of GtfC is currently available, thereby making it impossible to map the nine type I residues to the 3D structure of the enzyme to analyze their spatial distribution. Nonetheless, these nine residues represent attractive candidates for site-directed mutagenesis since they are likely involved in the functional divergence between GtfB and GtfC.

## Discussion

### Phylogenetic Perspective on the Evolution of Gtfs in Dental Plaque

Our phylogenetic reconstruction of the evolution of Gtfs shows that all the streptococcal glucosyltransferases form a robust monophyletic group, while the *Lactobacillus* and *Leuconostoc* glucosyltransferases are polyphyletic (Figure S1). Furthermore, our Bayesian analysis dates the common ancestor of streptococcal glucosyltransferases to 124–140 Mya (Figure 3), which roughly coincides with the divergence time of marsupials [32]. Streptococci have been shown to represent about 4% of the cultivable microbiota of dental plaque of marsupial species like kangaroos and wallabies [54].

The evidence lends itself to the speculation that an ancestral streptococcal species could have acquired a glucosyltransferase gene by lateral transfer around the time of divergence of viviparous, dentate animals, like marsupials and placental mammals. Streptococcal Gtfs synthesize two types of glucans: water-soluble dextran and water-insoluble mutan. Figures 1 and 3 suggest that the ancestral form of the streptococcal Gtfs likely synthesized dextran. Mutan has been strongly correlated with sucrose-dependent adherence in *S. mutans* [15], while dextran seems to play a minor role [11], mostly by aiding the adherence of insoluble glucans synthesized by other Gtfs [55] or by binding to other glucan-binding proteins in the cell surface [56]. Thus, the acquisition of a dextran-synthesizing Gtf by an ancestral streptococcal species may have contributed modestly to the colonization of the oral cavity, especially in the absence of WIG-synthesizing Gtfs.

On the other hand, the exploitation of the high energy glucosidic bond between the fructose and glucose molecules in sucrose, to produce an adhesive glucan (mutan) that mediated the attachment to the tooth surface would have conferred a greater selective advantage to the bacterium. This is supported by the observation that the dental plaque of modern humans is composed of 70% WIG, with only less than 2% of WSG [57]. Our data shows that the WIG-synthesizing streptococcal Gtfs, which seem predominant in the streptococcal species belonging mutans group, appeared around 85–90 Mya during the placental mammals lineage (Figure 3). The acquisition of a WIG-synthesizing Gtf likely promoted the colonization of the oral cavity by the ancestral streptococcal species.

The synthesis of WIG by *S. mutans* has long been recognized to be an important virulence factor in human dental caries [1], [58]. *S. mutans* strains isolated from humans harbor two genes encoding enzymes capable of synthesizing WIG, *gtfB* and *gtfC*. These two genes are highly homologous and arranged in tandem in the chromosome, which suggests that they arose by gene duplication. A chimeric *gtfBC* gene, likely the result of a recombination event between *gtfB* and *gtfC*, has been found in a small number of isolates, such as strain UA101 ([59], [60] and our unpublished results). One of the pieces of evidence that cemented the association between WIG and cariogenicity is that strain UA101 exhibits low levels of smooth caries activity in rats fed on a high-sucrose diet, which has been attributed to the reduced synthesis of WIG caused by this mutation [59], [60]. In a recent study of the ancestry of streptococcal Gtfs, Hoshino et al proposed that streptococci acquired glucosyltransferase genes by horizontal gene transfer when they encountered lactic acid bacteria present in fermented foods, and that the consumption of refined sugars by humans acted as a secondary selection pressure that prompted the acquisition of multiple *gtf* genes. Our results dissent with their model in that we found evidence of *gtfB*, *gtfC* and *gtfD* genes in the genomes of strains isolated from chimpanzees and macaques, animals that rarely manifest dental caries. Furthermore, the gene duplication event that gave rise to *gtfB* and *gtfC* took place approximately 46 to 53 Mya (Figure 3), around the time of divergence of early primates. Thus, the acquisition of GtfB and GtfC in *S. mutans* predates humans and does not coincide with the increased consumption of sucrose and other carbohydrates characteristic of the post-agricultural and post-industrial human diets [61]. After gene duplication, both duplicates can be maintained if their functions or expression patterns differ in some way [62]. GtfB and GtfC both synthesize WIG with 1,3-linked glucose as the major linkage, but the structure of the polymers is not identical. The glucans synthesized by GtfB and GtfC bound to saliva-coated hydroxyapatite beads display different branching points, and when the enzymes are in solution GtfC synthesizes a glucan with predominantly 1,6-linked glucose instead of 1,3-linked glucose like GtfB [63]. GtfB and GtfC also exhibit different sucrose-dependent binding properties [49], [4], [50], and their activities are different in a mixed oral biofilm model [51] and in response to starch hydrolysates [64]. The evidence suggests that GtfB and GtfC have acquired enough neofunctionalization for both copies to become fixed in the population.

The acquisition of two WIG-synthesizing Gtfs by gene duplication around the time of divergence of early primates was likely advantageous for the colonization and establishment of mutans streptococci in the oral cavity. The evidence suggests that the ancestral line leading to apes and humans was predominantly herbivorous, feeding mostly on dicotyledonous plants, fruits, flowers and leaves, with a minor intake of animal matter [65]. The small amount of sucrose present in wild-type fruits may have been sufficient to support colonization and establishment of mutans streptococci. Nevertheless, considerable evidence supports a direct relationship between dietary sucrose intake and plaque levels of mutans streptococci [66]. Thus, *S. mutans* likely benefitted from the increase in sucrose consumption in the human diet concomitant with the introduction of cultivated fruits and products containing refined sugar. This sets the stage for an intimate interaction between bacterium and host, with a central role for Gtfs.

### Correlates of Natural Selection to Catalytic Domains in Gtfs

We found evidence of negative selection on all three *S. mutans gtf* genes (Figure 2), particularly on the catalytic domain. This is in agreement with the finding by Ooshima and others [55] that all three enzymes are required in a particular ratio for optimal sucrose-dependent adhesion of *S. mutans* cells. Therefore, the seemingly small contribution of GtfD and soluble dextran to sucrose-dependent adherence may become more relevant in the presence of insoluble mutan synthesized by GtfB and GtfC.

The *gtfB* gene appeared to be the one under the strongest negative selection. This is consistent with the idea that the common ancestor to GtfB and GtfC was likely a WIG-synthesizing enzyme (Figure 1), and that after the gene duplication event the evolutionary constraints on GtfC were somewhat relieved, which allowed it to acquire the ability to also synthesize WSG. This was first postulated by Ueda and Kuramitsu [12] based on their finding of 24-bp homologous sequences flanking the *gtfB* and *gtfC* genes.

The statistically based approaches for detecting selection we employed have been criticized for not addressing functionality in a precise manner [67], [68]. The *S. mutans* Gtfs, however, display a high level of amino acid sequence identity (52% between GtfB/GtfC and GtfD and 74.5% between GtfB and GtfC), particularly in the catalytic domain. The high degree of conservation between these three enzymes allows for tests of hypotheses for functionality in the different domains.

The detection of type I functional divergence between GtfB and GtfC, but not of type II, suggests that they differ significantly in the site-specific evolutionary rates but not in site specific amino acid properties. Only two out of the nine residues identified as being critical for type I functional divergence are found in the catalytic domain (Figure 4). This is consistent with the high degree of conservation between the two enzymes and with the fact that both synthesize WIG. The position 540 of the alignment is occupied by a conserved aspartic acid in GtfB (Asp493), but that position is not conserved in GtfC or GtfD. Aspartic acid residues are present both in the active site and Gtf-P1 region of *S. mutans* Gtfs and their importance for the enzymatic activity has been shown by site-directed mutagenesis experiments [6], [69]. Moreover, the mutation of several other aspartic acid residues in GtfB led to the synthesis of glucans of increased solubility [70]. On the other hand, the position 566 in the alignment is not conserved in GtfB (Figure 4), but it is occupied by a conserved tyrosine residue in GtfC (Tyr545). GtfD also presents a conserved tyrosine residue at that position (Tyr533), suggesting that this residue might be relevant to the synthesis of WSG.

Five out of the nine residues identified as being critical for type I functional divergence between GtfB and GtfD are located in the C-terminal glucan-binding domain (GBD). The GBD of Gtfs is composed of a series of relatively conserved repeated units, known as YG repeats, responsible for the binding of glucan [6]. Nakano and Kuramitsu (1992) showed that the GBD influences the structure of the glucan synthesized by means of a fusion protein composed of the catalytic domain of GtfD (WSG) and the GBD of GtfB (WIG), which synthesized WIG in a primer-independent manner. The differences between the GBDs of GtfB and GtfC are expected to be subtler because of their common WIG-synthesizing activity. The residues identified by the functional divergence analysis are either hydrophilic (alignment positions 1339, 1402, 1422 and 1467, Figure 4) or hydrophobic (position 1456). The amino acid properties of those positions are unchanged between GtfB and GtfC, consistent with type I functional divergence, but the differences in evolutionary rate may hint to subtle differences in the hydrophobicity/hydrophilicity in the GBD instead. This is indeed confirmed by a hydropathy plot of the GBD of GtfB and GtfC ([71] Figure S2). The YG repeats are evidenced in the pattern of the hydropathy plot, which shows that GtfC presents stronger hydrophobic peaks than GtfB, as well as a C-terminal hydrophilic region (1417–1430) absent in GtfB. The biological significance of this region rich in polar amino acids may reside in the implication of polar residues in the creation of hydrogen bonds with hydroxyl residues of the sugar [6].

Our study constitutes an in-depth study of the evolution of streptococcal glucosyltransferases. We have provided evidence supporting the monophyly of this group that appears to cluster according to the type of glucan they synthesize, with the WIG-synthesizing Gtfs being found mostly in the mutans group. We also showed that the acquisition and diversification of *S. mutans* Gtfs predates humans and is therefore not associated with changes in human diet.

## Supporting Information

Figure S1 **Phylogenetic analysis of glucansucrases from lactic-acid bacteria.** ML tree of 66 glucansucrases based on full-length aminoacid sequences and the WAG+Γ+I+F model. Branch color indicates Red = *Streptococcus* spp.; Blue = *Leconostoc* spp.; Purple = *Lactobacillus* spp.; Brown = *Oenococcus oeni*; Magenta = *Weisella cibaria*.
(TIF)

Figure S2 **Kyte-Doolittle hydropathy plots of the GBD of** ***S. mutans*** **GtfB and GtfC.** The x-axis indicates the window position (window size = 7) and the y-axis the hydropathy score. Positive values indicate hydrophobic regions while negative values represent hydrophilic regions. The arrow indicates a highly hydrophobic region unique to GtfC.
(TIF)

Table S1 **Primers used in this study.**
(PDF)

Table S2 **Metadata for the sequences used in this study.**
(PDF)
