# Supplementary figures and images for: Phylogenetic Analysis of Glucosyltransferases and Implications for the Coevolution of Mutans Streptococci with Their Mammalian Hosts
Source: PLoS One. 2013 Feb 14;8(2):e56305. doi: 10.1371/journal.pone.0056305 (PMC3572963; doi:10.1371/journal.pone.0056305)

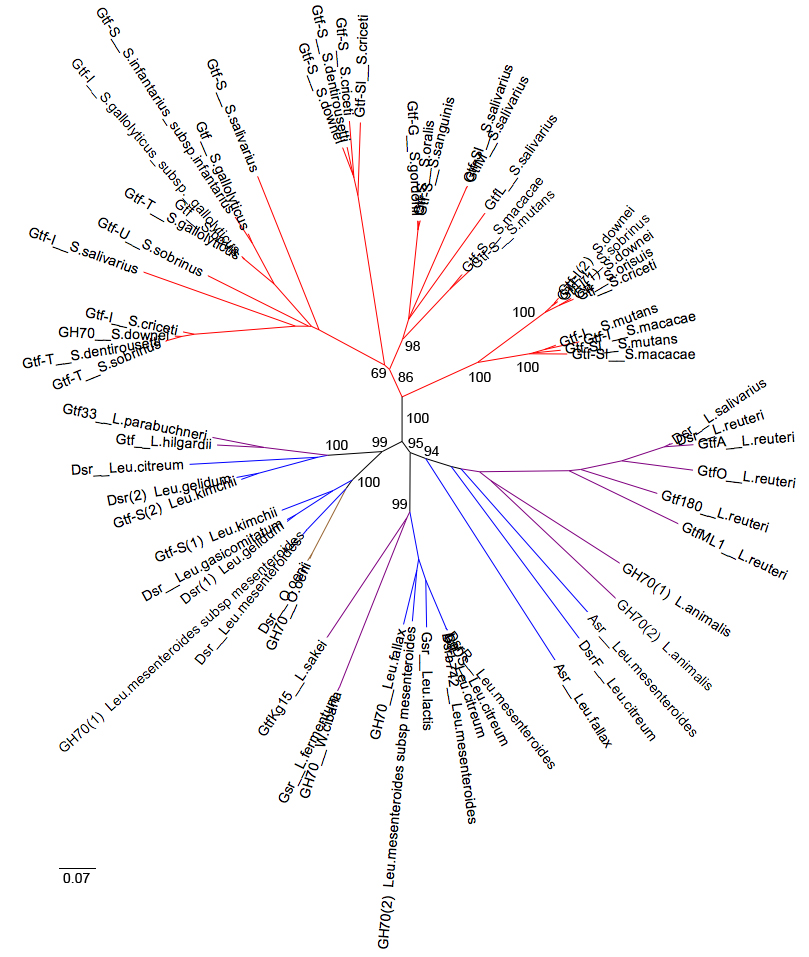

Supplement: Figure S1 — Phylogenetic analysis of glucansucrases from lactic-acid bacteria. ML tree of 66 glucansucrases based on full-length aminoacid sequences and the WAG+Γ+I+F model. Branch color indicates Red = Streptococcus spp.; Blue = Leconostoc spp.; Purple = Lactobacillus spp.; Brown = Oenococcus oeni; Magenta = Weisella cibaria. (TIF) [file pone.0056305.s001.tif]

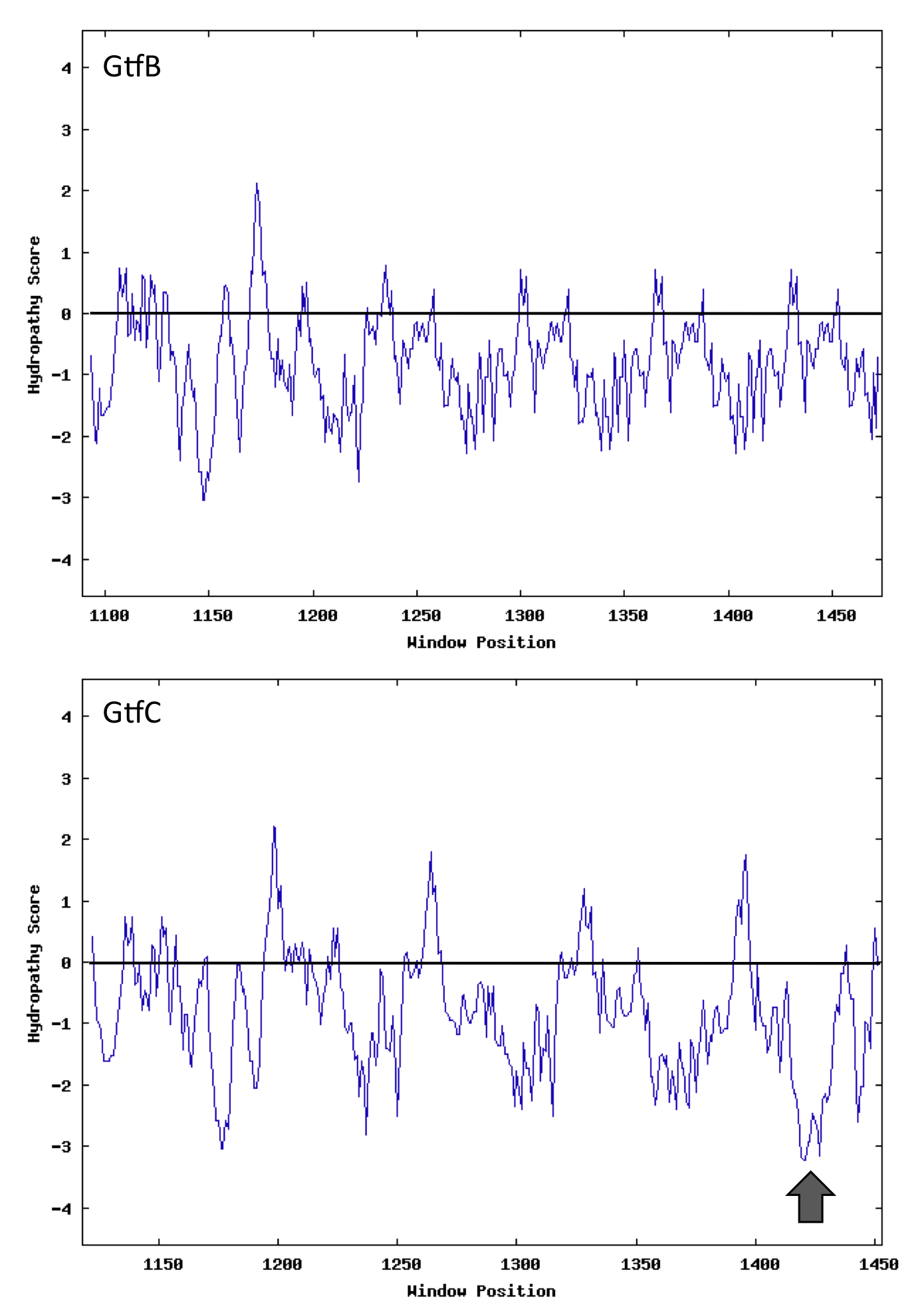

Supplement: Figure S2 — Kyte-Doolittle hydropathy plots of the GBD of S. mutans GtfB and GtfC. The x-axis indicates the window position (window size = 7) and the y-axis the hydropathy score. Positive values indicate hydrophobic regions while negative values represent hydrophilic regions. The arrow indicates a highly hydrophobic region unique to GtfC. (TIF) [file pone.0056305.s002.tif]
