# Supplementary material for: Phylogenetic Analysis of Glucosyltransferases and Implications for the Coevolution of Mutans Streptococci with Their Mammalian Hosts
Source: PLoS One. 2013 Feb 14;8(2):e56305. doi: 10.1371/journal.pone.0056305 (PMC3572963; doi:10.1371/journal.pone.0056305)
Supplement: Table S1 — Primers used in this study. (PDF) [file pone.0056305.s003.pdf]

Primer Name	Sequence (5'-3')	Use
GTF-001	ACGACAGCAATTAGACTGTTGTTT	PCR amplification of *gtfB (S. mutans)*
GTF-002	CTTTTCCATATTTCCTCCAAAAATAG	PCR amplification of *gtfB (S. mutans)*
GTF-003	CGTTACACTGCCATCAGCAA	PCR amplification of *gtfB* (*S. macacae*)
GTF-004	TTCTTTTCCATACATCCTCCAAAA	PCR amplification of *gtfB* (*S. macacae*)
GTF-005	ATGGAGCAGTGCTTTACGA	sequencing of *gtfB (S. mutans)*
GTF-006	TGCCATCAGCAATTAATGTA	sequencing of *gtfB (S. mutans)*
GTF-007	CTTCTGAAGCTGCTGCTAAT	sequencing of *gtfB (S. mutans)*
GTF-008	GGCAGAAATCAATCCTAATG	sequencing of *gtfB (S. mutans)*
GTF-009	AAGCGTAAGTTCCATCTTCA	sequencing of *gtfB (S. mutans)*
GTF-010	CACCATCTTCACCGAGATAC	sequencing of *gtfB (S. mutans)*
GTF-011	AGCCAAAGGAAAATTTGTAAC	sequencing of *gtfB (S. mutans)*
GTF-012	AGTTAATCCGAACTCGTTCTC	sequencing of *gtfB (S. mutans)*
GTF-013	AAAGCATAGCCGTTTTGG	sequencing of *gtfB (S. mutans)*
GTF-014	AAATGGAGCAGTGCTTTAC	sequencing of *gtfB (S. mutans)*
GTF-015	TAGTACGAACTTTGCCGT	sequencing of gtfB *(S. troglodytae)*
GTF-016	ATCTTTCTTTCCGGTTTGATT	sequencing of gtfB *(S. troglodytae)*
GTF-017	TATTTCTTCTCTGTAGCCAG	sequencing of gtfB *(S. troglodytae)*
GTF-018	TCATAACGATCCGTAAAGG	sequencing of gtfB *(S. troglodytae)*
GTF-019	AATGTGGATAAGTTTGCGG	sequencing of gtfB *(S. troglodytae)*
GTF-020	GGCGTCACTTTAATAATGGT	sequencing of gtfB *(S. troglodytae)*
GTF-021	GTAATGATTTCAGAATTGCCA	sequencing of gtfB *(S. troglodytae)*
GTF-022	CTCACTGTTCCAAGCTGA	sequencing of gtfB *(S. troglodytae)*
GTF-023	AGCGAAAAACCGTTTGAT	sequencing of gtfB *(S. troglodytae)*
GTF-024	CAGAGACAAACAACAAGCAA	sequencing of *gtfB* (*S. macacae*)
GTF-025	GTTTTTCGCTTTCACTGTTC	sequencing of *gtfB* (*S. macacae*)
GTF-026	TTAATGGTAAGGCTCTGCAT	sequencing of *gtfB* (*S. macacae*)
GTF-027	AGCATCTACACGAATGGAGT	sequencing of *gtfB* (*S. macacae*)
GTF-028	GAACAGTGAAAGCGAAAAAC	sequencing of *gtfB* (*S. macacae*)
GTF-029	GACGACTTGTTCATTTTCGT	sequencing of *gtfB* (*S. macacae*)
GTF-030	GCATGAATCCTTTGATCACT	sequencing of *gtfB* (*S. macacae*)
GTF-031	TCTGCCGTACCGTATTTATT	sequencing of *gtfB* (*S. macacae*)
GTF-032	GCGAGTGAAACTCCATCTAC	sequencing of *gtfB* (*S. macacae*)
GTF-033	ACCAGTCACCATGTAACCAT	sequencing of *gtfB* (*S. macacae*)
GTF-034	TCAGGAAAAGATCAACAAGC	sequencing of *gtfB* (*S. macacae*)
GTF-035	TAATAGCTGATACGGCCTTG	sequencing of *gtfB* (*S. macacae*)
GTF-036	AAGAATCTGGCAATATGCAC	sequencing of *gtfB* (*S. macacae*)
GTF-037	TACCTTACCAGCTTCATCGT	sequencing of *gtfB* (*S. macacae*)
GTF-038	AGGGCGTTTCTAGGGTTAGG	PCR amplification of *gtfC (S. mutans)*
GTF-039	CAAGGCATGCGTCTCTTCTT	PCR amplification of *gtfC (S. mutans)*
GTF-040	GCCGTATCAGGATGACAATCA	PCR amplification of *gtfC* (*S. macacae)*
GTF-041	CAAGACCAAAGTCCTGCCTCT	PCR amplification of *gtfC* (*S. macacae)*
GTF-042	CGATGCTAACTCTGGAGAACG	PCR amplification of gtfC *(S. troglodytae)*
GTF-043	TTTTAGTTTGGCTCTGCCTTTTT	PCR amplification of gtfC *(S. troglodytae)*
GTF-044	TTCTGTTGACTGTGTCCATG	sequencing of *gtfC (S. mutans)*
GTF-045	TGGGAAAACTTTCTTCTTTG	sequencing of *gtfC (S. mutans)*
GTF-046	CTTCACAGGCTAATTCCAAC	sequencing of *gtfC (S. mutans)*
GTF-047	GAAGGCTTTCGAGATTTACA	sequencing of *gtfC (S. mutans)*
GTF-048	TAGCTTGGTTACCACTCGTT	sequencing of *gtfC (S. mutans)*

GTF-049	ATCAAAGTAACGCAGCTTTC	sequencing of *gtfC (S. mutans)*
GTF-050	CGTCGTTATGAAAATGGTTAC	sequencing of *gtfC (S. mutans)*
GTF-051	TTTTCATCAAAGTAAACACGTC	sequencing of *gtfC (S. mutans)*
GTF-052	TCTCTTTCAGGTGGATTTGT	sequencing of *gtfC* (*S. macacae*)
GTF-053	GCCACCAGGACATAAGAATA	sequencing of *gtfC* (*S. macacae*)
GTF-054	ACAAAGAAGATGGCACTGTT	sequencing of *gtfC* (*S. macacae*)
GTF-055	TCTTTCCAAAATTCATGAGG	sequencing of *gtfC* (*S. macacae*)
GTF-056	CAAGTATGGGAGGCTATGAG	sequencing of *gtfC* (*S. macacae*)
GTF-057	CGTATTTTATCCGTGCTTTC	sequencing of *gtfC* (*S. macacae*)
GTF-058	CTGAAATCAATCCCAATGTT	sequencing of *gtfC* (*S. macacae*)
GTF-059	CAAAATCTTGGAAATTCGAG	sequencing of *gtfC* (*S. macacae*)
GTF-060	CTCGTTTATCAGGAGTTGCT	sequencing of *gtfC* (*S. macacae*)
GTF-061	CCGTTGAAATCTGTTTCCTA	sequencing of *gtfC* (*S. macacae*)
GTF-062	AGATGGGACTTTCCTTGATT	sequencing of *gtfC* (*S. macacae*)
GTF-063	TGACAGTCGTTAAACCAACA	sequencing of *gtfC* (*S. macacae*)
GTF-064	ATGACGGGAAAGGCTATACT	sequencing of *gtfC* (*S. macacae*)
GTF-065	TAGTATTTGACACGGCCTTT	sequencing of *gtfC* (*S. macacae*)
GTF-066	TCCAAGCTAAAGGACAGTTT	sequencing of *gtfC* (*S. macacae*)
GTF-067	CATGCTAATTCCAACTACCG	sequencing of gtfC *(S. troglodytae)*
GTF-068	GGAGCAGGCTATGTCTTAAA	sequencing of gtfC *(S. troglodytae)*
GTF-069	GACCGCCTGAAACATACTT	sequencing of gtfC *(S. troglodytae)*
GTF-070	ACCAGTGACCATATAGCCG	sequencing of gtfC *(S. troglodytae)*
GTF-071	ATGCAGCTTTCCATCAGC	sequencing of gtfC *(S. troglodytae)*
GTF-072	CCAAGCTTTCGCCACTAAAA	sequencing *gtfB* and *gtfC (S. mutans)*
GTF-073	GAATCAAAGTTAGCATCCG	sequencing *gtfB* and *gtfC (S. mutans)*
GTF-074	GCC ATT ACC TTA ATG CCC T	sequencing *gtfB* and *gtfC (S. mutans)*
GTF-075	GATCGTCTTATGAGCCATGT	sequencing *gtfB* and *gtfC (S. mutans)*
GTF-076	AATGGGACAAATATTTTAGGG	sequencing *gtfB* and *gtfC (S. mutans)*
GTF-077	TTATTTTACGACGGTTGTTGT	PCR amplification of *gtfD (S. mutans)*
GTF-078	AAAGCCTGACTGGAAGCAGT	PCR amplification of *gtfD (S. mutans)*
GTF-079	TTTTACGCGAGCCTTAATAGA	PCR amplification of gtfD *(S. troglodytae)*
GTF-080	AGAAGTATCATTTCCCTGAGC	PCR amplification of gtfD *(S. troglodytae)*
GTF-081	CATGGCCTTAGCCAGTTTATT	PCR amplification of *gtfD* (*S. macacae*)
GTF-082	CAGCGGCTGTTTGTACTTGA	PCR amplification of *gtfD* (*S. macacae*)
GTF-083	GCAAACGTTGCTGATACAAG	sequencing of *gtfD (S. mutans)*
GTF-084	GTTTGCTGCGTTTGAGAAAT	sequencing of *gtfD (S. mutans)*
GTF-085	TAATGTCTTGGTGGCCAGAT	sequencing of *gtfD (S. mutans)*
GTF-086	GCGATAGGCGCAGTTTATTA	sequencing of *gtfD (S. mutans)*
GTF-087	AAAGCCCATTATGGTGTTGA	sequencing of *gtfD (S. mutans)*
GTF-088	AATGACAGCCATTCCTTGTG	sequencing of *gtfD (S. mutans)*
GTF-089	CGGTGGTCAAGACATGAAG	sequencing of *gtfD (S. mutans)*
GTF-090	TCTTGCTGAGAACCGTATTTG	sequencing of *gtfD (S. mutans)*
GTF-091	TCAAATCTTGGGGTGTCACT	sequencing of *gtfD (S. mutans)*
GTF-092	AGATGCTGCAAGCCATAAAC	sequencing of *gtfD (S. mutans)*
GTF-093	TTTGTTAATGATGGCAATGG	sequencing of *gtfD (S. mutans)*
GTF-094	TCGCTTGCAAAATAGAGCTT	sequencing of *gtfD (S. mutans)*
GTF-095	TATTTCCTTGCCAATTCAGG	sequencing of *gtfD (S. mutans)*
GTF-096	CCATTATCGGTCGGTTTGT	sequencing of *gtfD (S. mutans)*
GTF-097	TGATGACAAAACGGTGACAG	sequencing of gtfD *(S. troglodytae)*

GTF-098	TTGGCTGCGTTTTAACAAA	sequencing of gtfD *(S. troglodytae)*
GTF-099	ACTGGTAATGACAGCCATACC	sequencing of gtfD *(S. troglodytae)*
GTF-100	GCGTTTTGACTTCTGTTCGT	sequencing of gtfD *(S. troglodytae)*
GTF-101	CAATCCGCAATGACTTGAAT	sequencing of gtfD *(S. troglodytae)*
GTF-102	CTTTGGTCAAGATGGCAAGC	sequencing of gtfD *(S. troglodytae)*
GTF-103	TAATGGCGACAGGTATTTCC	sequencing of gtfD *(S. troglodytae)*
GTF-104	AAAGGCAGTTCAGCACAGGT	sequencing of *gtfD* (*S. macacae*)
GTF-105	TCGCCAGCTGAAGTATCAGA	sequencing of *gtfD* (*S. macacae*)
GTF-106	TGCAGCTCAAACTGTCCAAG	sequencing of *gtfD* (*S. macacae*)
GTF-107	CGCTGGCTGTTTTTACCTTC	sequencing of *gtfD* (*S. macacae*)
GTF-108	AGGTGCACAGCTCCCTATTG	sequencing of *gtfD* (*S. macacae*)
GTF-109	GCCGCCCCCATATTAACTAC	sequencing of *gtfD* (*S. macacae*)
GTF-110	CGCTATGGTACAGGGGCTAA	sequencing of *gtfD* (*S. macacae*)
GTF-111	GATCTGATCTGGCACCCAGT	sequencing of *gtfD* (*S. macacae*)
GTF-112	GGCACCGCAATACGTATCAT	sequencing of *gtfD* (*S. macacae*)
GTF-113	AACGCCATTTTGCAAGAAATA	sequencing of *gtfD* (*S. macacae*)
GTF-114	TGATGATGGCAAGGGAATTA	sequencing of *gtfD* (*S. macacae*)
GTF-115	CTGCCTTTCACTTGCTTTCC	sequencing of *gtfD* (*S. macacae*)
GTF-116	TTTTTGCCACAGACAGTCAAA	sequencing of *gtfD* (*S. macacae*)
GTF-117	GCTTCGTTGCCTTACTGCTAA	sequencing of *gtfD* (*S. macacae*)
