# Supplementary material for: Phylogenetic Analysis of Glucosyltransferases and Implications for the Coevolution of Mutans Streptococci with Their Mammalian Hosts
Source: PLoS One. 2013 Feb 14;8(2):e56305. doi: 10.1371/journal.pone.0056305 (PMC3572963; doi:10.1371/journal.pone.0056305)
Supplement: Table S2 — Metadata for the sequences used in this study. (PDF) [file pone.0056305.s004.pdf]

GI Accession	Locus Accession	Definition	Organism	Strain	Gene Name	Origin, site	Protein Length	Type of Glucan
335358117	ZP_08549987.1	glycosyl hydrolase family 70 protein	Lactobacillus animalis	KCTC 3501		Baboon, oral	1651	N/F
335358118	ZP_08549988.1	glycosyl hydrolase family 70 protein	Lactobacillus animalis	KCTC 3501		Baboon, oral	1573	N/F
51574164	AAU08008.1	glucansucrase	Lactobacillus fermentum	Kg3	gtfKg3	N/F	1463	Dextran
301128693	CBJ19544.1	glucosyltransferase (dextransucrase)	Lactobacillus hilgardii	TMW 1.828		Water kefir	1448	Dextran
51574161	AAU08006.1	glucansucrase	Lactobacillus parabuchneri	33	gtf33	N/F	1561	Dextran
51574154	AAU08001.1	glucansucrase	Lactobacillus reuteri	180	gtf180	N/F	1772	Dextran
62736384	AAX97502.1	glucansucrase	Lactobacillus reuteri	ATCC 55730	gtfO	Human, breast milk	1781	Reuteran
51574173	AAU08015.1	glucansucrase	Lactobacillus reuteri	121	gtfA	Pig, intestine	1781	Reuteran
145666440	ABP88726.1	dextransucrase	Lactobacillus reuteri	TMW1.106	gtf106A	Sourdough	1782	N/F
51574158	AAU08004.1	glucansucrase	Lactobacillus reuteri	ML1	gtfML1	Mouse	1772	Mutan
51574168	AAU08011.1	glucansucrase	Lactobacillus sakei	Kg15	gtfKg15	N/F	1595	Dextran
335350707	EGM52203.1	dextransucrase	Lactobacillus salivarius	GJ-24		Human, intestine	1786	N/F
164454297	BAF96719.1	dextransucrase	Leuconostoc citreum	HJ-P4 (KACC 91035)	LcDS	Kimchi	1477	Dextran
296470401	ACY92456.2	dextransucrase	Leuconostoc citreum	B/110-1-2	dsrF	Sugar-cane juice	1527	Dextran
170016491	YP_001727410.1	glucosyltransferase	Leuconostoc citreum	KM20		Kimchi Baechu	1505	N/F
170017743	YP_001728662.1	dextransucrase	Leuconostoc citreum	KM20		Kimchi Baechu	1495	N/F
330717998	ZP_08312598.1	alternansucrase	Leuconostoc fallax	KCTC 3537		Sauerkraut	1874	N/F
330718209	ZP_08312809.1	glycosyl hydrolase family 70 protein	Leuconostoc fallax	KCTC 3537		Sauerkraut	1543	N/F
300173313	YP_003772479.1	dextransucrase	Leuconostoc gasicomitatum	LMG 18811	dsrA	Marinated broiler meat	1526	N/F
333398048	ZP_08479861.1	dextransucrase	Leuconostoc gelidum	KCTC 3527		Kimchi	1528	N/F
333398151	ZP_08479964.1	dextransucrase	Leuconostoc gelidum	KCTC 3527		Kimchi	1422	N/F
296112122	YP_003622504.1	glucosyltransferase-S	Leuconostoc kimchii	IMSNU 11154		Kimchi	1549	N/F
296111841	YP_003622223.1	glucosyltransferase-S	Leuconostoc kimchii	IMSNU 11154		Kimchi	1455	N/F
251825496	ACT20911.1	glucansucrase	Leuconostoc lactis	EG001		Kimchi	1500	N/F
45934744	AAS79426.1	dextransucrase	Leuconostoc mesenteroides	IBT-PQ	dsrP	Pulque	1454	Dextran
10862851	CAB65910.2	alternansucrase	Leuconostoc mesenteroides	NRRL B-1355	asr	Soil	2057	Alternan
29465860	AAN38835.1	dextransucrase	Leuconostoc mesenteroides	NRRL B-1501	dsrR	Sugar-cane juice	1330	N/F
11559649	AAG38021.1	dextransucrase	Leuconostoc mesenteroides	B-742CB	dsrb742	N/F	1508	Dextran
116618836	YP_819207.1	glycosyl hydrolase family 70 protein	Leuconostoc mesenteroides subsp. mesenteroides	ATCC 8293		Fermenting olives	1527	N/F
116618841	YP_819212.1	glycosyl hydrolase	Leuconostoc mesenteroides subsp. mesenteroides	ATCC 8293		Fermenting olives	1514	N/F
118586951	ZP_01544383.1	dextransucrase	Oenococcus oeni	ATCC BAA-1163	dsrT	Bordeaux wine	1100	N/F
116490611	YP_810155.1	glycosyl hydrolase	Oenococcus oeni	PSU-1		Wine	1475	N/F
146741366	BAF62338.1	glucosyltransferase-I	Streptococcus criceti	GTC242 (HS-6)	gtfI	Hamster, oral	1461	Mutan
342837857	ZP_08711720.1	glucosyltransferase-S2	Streptococcus criceti	GTC242 (HS-6)	gtfS2	Hamster, oral	1512	N/F
357235604	ZP_09122947.1	glucosyltransferase-S1	Streptococcus criceti	GTC242 (HS-6)	gtfS1	Hamster, oral	1338	N/F
357236477	ZP_09123820.1	glucosyltransferase-SI1	Streptococcus criceti	GTC242 (HS-6)	gtfSI1	Hamster, oral	1393	N/F
356885157	EHI75357.1	glucosyltransferase-SI2	Streptococcus criceti	GTC242 (HS-6)	gtfSI2	Hamster, oral	1542	N/F
167735926	BAG07220.1	glucosyltransferase-I	Streptococcus dentirousetti	NUM1302	gtfI	Bat, oral cavity	1466	Mutan
358350618	BAL15792.1	glucosyltransferase-S	Streptococcus dentirousetti	NUM1302	gtfS	Bat, oral	1369	N/F
358350620	BAL15793.1	glucosyltransferase-T	Streptococcus dentirousetti	NUM1302	gtfT	Bat, oral	1508	Dextran
146741368	BAF62339.1	glucosyltransferase-I	Streptococcus dentisuis	NUM1103	gtfI	Pig, oral cavity	1466	N/F
153652	AAA26898.1	glucosyltransferase-S	Streptococcus downei	MFe28 (ATCC 33748)	gtfS	Macaque, oral	1365	Dextran
121724	P11001.1	glucosyltransferase-I	Streptococcus downei	MFe28 (ATCC 33748)	gtfI	Macaque, oral	1597	Mutan
312865974	ZP_07726195.1	KxYKxGKxW signal domain protein	Streptococcus downei	F0415		Human, oral	1505	N/F
156563506	BAF76048.1	glucosyltransferase	Streptococcus equinus	ATCC 33317	gtf	Cow, dung	1546	N/F
288905256	YP_003430478.1	glucosyltransferase	Streptococcus gallolyticus	UCN34	gtfA	Human, endocarditis	1522	N/F
288905258	YP_003430480.1	glucosyltransferase-T	Streptococcus gallolyticus	UCN34	gtfB	Human, endocarditis	1412	N/F
325978242	YP_004287958.1	glucosyltransferase-I	Streptococcus gallolyticus subsp. gallolyticus	ATCC BAA-2069	gtfB1	Human, blood	1545	N/F
157150042	YP_001449813.1	glucosyltransferase G	Streptococcus gordonii	ATCC 35105	gtfG	Human, oral	1576	Dextran
171779392	ZP_02920356.1	hypothetical protein STRINF_01237	Streptococcus infantarius subsp. infantarius	ATCC BAA-102	gtfD?	Human, feaces	1448	N/F
357584985	EHJ52188.1	glucosyltransferase-S	Streptococcus macacae	NCTC 11558	STRMA_0560	Macaque, oral	1471	N/F
357584774	EHJ51977.1	glucosyltransferase-I	Streptococcus macacae	NCTC 11558	STRMA_0629	Macaque, oral	1418	N/F
357585503	EHJ52706.1	glucosyltransferase-SI	Streptococcus macacae	NCTC 11558	STRMA_0630	Macaque, oral	1448	N/F
24379444	NP_721399.1	glucosyltransferase-I	Streptococcus mutans	UA159	gtfB	Human, oral	1476	Mutan
24379445	NP_721400.1	glucosyltransferase-SI	Streptococcus mutans	UA159	gtfC	Human, oral	1455	Mutan, Dextran
24379358	NP_721313.1	glucosyltransferase-S	Streptococcus mutans	UA159	gtfD	Human, oral	1462	Dextran
293365092	ZP_06611809.1	glucosyltransferase-S	Streptococcus oralis	ATCC 35037		Human, oral	1575	Dextran
146741364	BAF62337.1	glucosyltransferase-I	Streptococcus orisuis	NUM 1001 (JCM14035)	gtfI	Pig, oral	1466	Dextran
153649	AAA26896.1	glucosyltransferase-I	Streptococcus salivarius	ATCC 25975	gtfJ	Human, oral	1518	Mutan
47531	CAA77898.1	glucosyltransferase-S	Streptococcus salivarius	ATCC 25975	gtfK	Human, oral	1599	Dextran
662379	AAC41412.1	glucosyltransferase	Streptococcus salivarius	ATCC 25975	gtfL	Human, oral	1449	Mutan
662381	AAC41413.1	glucosyltransferase	Streptococcus salivarius	ATCC 25975	gtfM	Human, oral	1577	Dextran
228476661	ZP_04061337.1	glucosyltransferase-SI	Streptococcus salivarius	SK126		Human, skin	1569	N/F
325688181	EGD30200.1	glucosyltransferase-S	Streptococcus sanguinis	SK72		Human, oral	1575	N/F
121725	P27470.1	glucosyltransferase-I	Streptococcus sobrinus	6715	gtfI	Human, oral	1592	Mutan
62199787	AAX76986.1	glucosyltransferase-S2	Streptococcus sobrinus	B13N	gtfT	N/F	1506	Dextran
22138845	BAC07265.1	glucosyltransferase-S1	Streptococcus sobrinus	B13N	gtfU	N/F	1554	Dextran
940427	BAA09792.1	glucosyltransferase-I	Streptococcus sobrinus	ATCC 33478 (SL1)	gtfI	Human, oral	1590	Mutan
332638569	ZP_08417432.1	glycosyl hydrolase family 70 protein	Weissella cibaria	KACC 11862		Kimchi	1448	N/F
